# A Novel Energy-Efficient Multi-Sensor Fusion Wake-Up Control Strategy Based on a Biomimetic Infectious-Immune Mechanism for Target Tracking

**DOI:** 10.3390/s18041255

**Published:** 2018-04-18

**Authors:** Jie Zhou, Yan Liang, Qiang Shen, Xiaoxue Feng, Quan Pan

**Affiliations:** 1 School of Automation & MOE Key Laboratory of Information Fusion Technology, Northwestern Polytechnical University, Xi’an 710072, China; zhoujie@mial.nwpu.edu.cn (J.Z.); quanpan@nwpu.edu.cn (Q.P.); 2 MOE Key Laboratory of Micro and Nano Systems for Aerospace, Northwestern Polytechnical University, Xi’an 710072, China; shenq@nwpu.edu.cn; 3 School of Automation, Beijing Institute of Technology, Beijing 100081, China; fengxiaoxue@bit.edu.cn

**Keywords:** immune mechanism, infectious disease, energy efficiency, wake-up strategy, multi-sensor fusion

## Abstract

A biomimetic distributed infection-immunity model (BDIIM), inspired by the immune mechanism of an infected organism, is proposed in order to achieve a high-efficiency wake-up control strategy based on multi-sensor fusion for target tracking. The resultant BDIIM consists of six sub-processes reflecting the infection-immunity mechanism: occurrence probabilities of direct-infection (DI) and cross-infection (CI), immunity/immune-deficiency of DI and CI, pathogen amount of DI and CI, immune cell production, immune memory, and pathogen accumulation under immunity state. Furthermore, a corresponding relationship between the BDIIM and sensor wake-up control is established to form the collaborative wake-up method. Finally, joint surveillance and target tracking are formulated in the simulation, in which we show that the energy cost and position tracking error are reduced to 50.8% and 78.9%, respectively. Effectiveness of the proposed BDIIM algorithm is shown, and this model is expected to have a significant role in guiding the performance improvement of multi-sensor networks.

## 1. Introduction

With the advantages of being low-cost, easy to implement, self-organizing, and highly reliable, multi-sensor fusion networks are widely used for such applications as non-cooperative tracking [1,2], forest fire detection [3], industrial process control [4], water quality detection [5], and machine health monitoring [6], and object state detection [7,8]. These sensor networks are composed of a group of cooperating low-power, low-precision, inexpensive sensor nodes equipped with limited transmission range transceivers, a small data processing unit, constrained memory, and limited available energy. However, due to dense sensor node deployment and the infrequent occurrences of the targets for each sensor node, too much energy will be consumed when the targets are absent if wake-up control is not utilized [9,10]. The fact that power consumption constraints exist for sensor nodes using un-rechargeable batteries [11] means that it is essential to balance conflicting performance requirements adaptively between energy consumption and target sensing.

The most widely used technique for lifetime optimization of multi-sensor networks is sensor wake-up control (WC) [12], which is used to adaptively determine the “wake up” or “sleep” states of each node. A “wake up” sensor node is in charge of collecting and processing the measurements and transferring them to its neighbors or fusion center, while a “sleep” sensor node closes its sensing, communication, and processing modules for energy saving purposes. Currently, two different research methods are employed improve the energy efficiency of the WC of multi-sensor networks. The first mainly focuses on designing different channel assignments [13,14,15], power controls [16], sampling rates [17], and MAC/routing protocols [13,14,18]. These methods are usually classified as being in the wireless communication research field of sensor networks, which mainly emphasizes the back-end network protocol design of the system. The second focus of research is primarily interested in establishing different models of multi-sensors themselves and developing corresponding control algorithms. These research methods usually belong to the information fusion field of sensor networks, which theoretically emphasizes the front-end sensor model and fusion algorithm exploration of the system. Further, for the WC strategies viewed from the information fusion field, classical WC scheduling strategies can be divided into three categories: surveillance-oriented, tracking-oriented, and topology-oriented. In the surveillance-oriented category, all sensor nodes are set to randomly “sleep” and “wake” independently, with the aim of achieving homogeneous wide-area coverage with even energy consumption [19,20]. For the tracking-oriented WC method, the sensor nodes in the vicinity of the predicted target location are woken up successively along the predicted target track, and, during this process, one or more clusters are formed, and one sensor node will be chosen as the cluster leader in each cluster, which is in charge of gathering target information and controlling the nodes’ states [21,22,23,24,25]. The authors of [22] utilized a PSO algorithm and duty cycling technology in order to let the selected sensors perform tasks. The authors of [23] proposed a sensor selection strategy based on a multi-objective optimization method to save energy. However, the performance of this method is restricted by localization accuracy of the sensor nodes, as low node localization will lead to poor target positioning and therefore improper wake up clusters. The third category is the topology-oriented WC strategy [26,27,28,29,30,31,32], which gathers nodes together to form a cluster, in which the node states of “wake” and “sleep” change dynamically. The elected cluster leader keeps the “wake” state to detect the target. A new cluster with two heads was proposed in [27] to track the targets collaboratively to enhance the robustness and network lifetime. In [28], two distributed information fusion schemes are proposed in anti-submarine warfare applications. In [29], an energy-efficient adaptive overlapping clustering method is proposed for continuous monitoring applications. In [30], a strategy is proposed to track a moving target in an environment with obstacles. This cluster leader will wake up its members once a target is found. Therefore, the decisions regarding target detecting and tracking are dominated completely by a single node. This will inevitably lead to a number of false alarms.

Recently, an artificial ant colony (AAC) approach was proposed to allow distributed sensor WC in WSN to accomplish the joint task of surveillance and target tracking, which in ants is then transformed into information on food location [9]. The communication, invalidation, and fusion of the target information are modeled as the processes of pheromone diffusion, loss, and accumulation, respectively. Compared with classical wake-up methods, a similar position error is obtained by the AAC method with much lower energy consumption, indicating that swarm intelligence is promising in dealing with the problem of distributed WC in WSNs. However, in the AAC method, the information is modeled as the pheromones that ants release, so whether the information comes from a sensing module (SM) or a communication module (CM) is not distinguishable. Therefore, as in classical wake-up methods, the AAC method is limited to optimizing the energy consumption of the SMs. However, in dynamic power management techniques [33], the energy consumed by the CM cannot be ignored, which is why we presented a distributed infectious disease model (DIDM) in a prior work [34]. With four sub-processes and six commonsense rules, the information from the SM and CM was modeled as the pathogen spread by direct infection and cross-infection, respectively. The simulation shows that the DIDM method is more effective than the AAC approach.

On the other hand, the DIDM method placed most emphasis on the infection mechanism. For the immune mechanism, it only introduces a prior identical immunity repairing ratio 0 < *q* < 1, ignoring the variation tendency in the infectious process. However, in practice, with changes in infection, the biological immune capacity changed correspondingly. Therefore, it is reasonable to design the immune mechanism according to natural processes and dynamical behavior in the biological immune system. With such an immune mechanism, the model deficiency caused by the fixed immunity repairing ratio can be avoided. Motivated by this, a biomimetic distributed infection-immunity model (BDIIM) that includes three sub-processes related to the infection mechanism—the occurrence probabilities of direct-infection and cross-infection, the immunity/immune-deficiency of direct-infection and cross-infection, and the pathogen amount of direct-infection and cross-infection—is proposed. In addition, two sub-processes—immune cell production and immune memory—are added to depict the immune mechanism, and one sub-process—pathogen accumulation under immunity state—is revised. Furthermore, the relationship between sensor wake-up and the BDIIM is established to derive a collaborative wake-up method. A simulation of joint surveillance and target tracking is utilized to show the effectiveness of BDIIM.

The rest of the paper is organized as follows: Section 2 establishes the BDIIM for node wake-up control and proposes a BDIIM model, and Section 3 establishes the correspondence between sensor wake-up and disease propagation. The proposed method is evaluated and analyzed by simulations in Section 4. Finally, Section 5 concludes the paper.

## 2. Establishment of a BDIIM for Node Wake-Up Control

### 2.1. Wake-Up Problem Description

**Figure 1:**
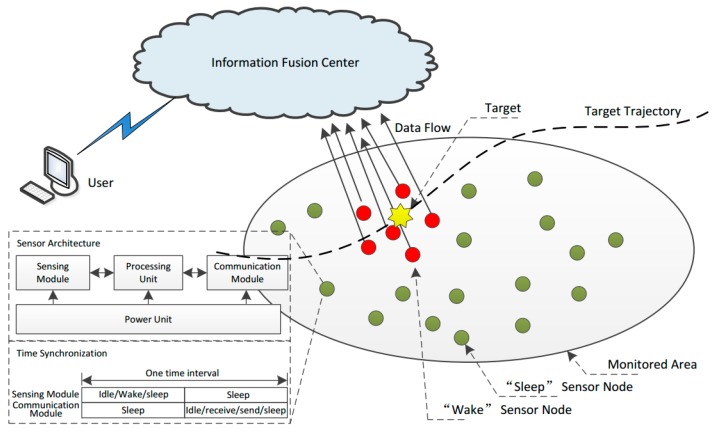
The problem formulation of sensor wake-up and architecture of a sensor node.

The problem formulation is shown in Figure 1. The target is continuously moving within a surveillance zone. Each sensor is equipped with one sensing module (SM) and one communication module (CM) and, in each time interval, the sensors are randomly in a “wake” or “sleep” state. In the “wake” state, the SM is powered on and monitors its working area. If one sensor finds the target, then the corresponding sensor will inform its neighbors through the CM. In each time interval, all of the “wake” sensors, which find the target, report their locations to the information fusion center, then the fusion center will obtain the target’s location and pass the location information to the users. After the target has passed by, the sensors do not sense the target anymore and will be in a sleeping state again. The goal of this section is to adaptively and distributively wake up the SM and CM of each node in each time interval so as to obtain intensive node coverage of the target with low energy consumption in joint target surveillance and tracking. Consider a sensor network consisting of N nodes uniformly distributed in a two-dimensional monitoring area, which satisfies the following assumptions:

(1) All the sensor nodes are stationary on the ground and each one is assigned a unique ID. Each sensor node knows its location via a GPS module or other localization techniques.
(2) The target is non-cooperative in maneuvering and its movement model is unknown. When and where it appears and disappears, and the length of time of its appearance, are also random and unknown.
(3) All the sensors in the network are binary detectors and equipped with an SM of one identical fixed sensing region (a disk area centered at the node with a radius *R_s_*). In each time instant, the power-on sensor node outputs “1” when it detects targets presenting in its sensing region, or outputs “0” when it does not detect any target. The detection probability is *P_d_*, which denotes the probability that the node detects the target while the target is actually within its sensing region. The false-alarm probability is *P_f_*, which denotes the probability it detects a target while there is no target within its sensing region.
(4) All the sensors in the network are equipped with a CM with an identical fixed communicate zone (a disk area centered at the node with the radius being *R_c_*). Sensors within the communicate zone are considered neighbors of the corresponding sensor. When a sensor node detects targets within its sensing region, it will inform its neighbors. In general, the node communication range is equal to the one hop communication distance, so any adjacent nodes can directly exchange their information without data routing.
(5) All the sensor nodes maintain time synchronization, and we choose a one round-based synchronization protocol, such as the Sensor Medium-Access Control (S-MAC) protocol [17]. In such protocols, as shown in Figure 1, the SM and CM work in turn. In each time interval, when the SM is in operation mode (including three phases: “idle/wake/sleep”), it randomly switches among the three phases according to its waking probabilities. The “sleep” SM stays powered off, while the “idle/wake” SM is powered on. The difference between “idle” and “wake” is that the former is in charge of target detection while the latter does nothing. When the CM is in operation mode, it randomly switches among four phases according to the CM wake-up probabilities. Like the SM, “sleep” and “idle” also means “powered off” and “powered on but doing nothing”. The “send/receive” mode means such a CM sends or receives messages to or from its neighbors.

### 2.2. Modeling of the Biomimetic Infectious-Immune Process

#### 2.2.1. Immune Mechanism

Due to widely well-known immune mechanism [35,36,37], corresponding immune characteristic is just described briefly in this work as follows: The immune system provides protections for the organisms from infection diseases. The immune system is capable of generating a specific immune response against the invading pathogen to control the infection and retains the memory of this pathogen. Organisms have the ability to mount an accelerated immune response upon re-exposure to the same pathogen. This rapid recall response can either completely prevent disease or greatly lessen the severity of clinical symptoms [38]. For example, smallpox vaccine-specific memory B cells last more than 50 years in immunized individuals [39], while the immune memory of measles can be detected up to 34 years after vaccination [40].

#### 2.2.2. Previous Distributed Infectious Disease Model

Our previous DIDM is designed based on the infection mechanism. In this section, we briefly describe the DIDM. Before the description, some concepts must be listed first.

- A pathogen refers to a microorganism that may make humans or animals sick. Such a pathogen may be transmitted to other individuals within a certain distance. An immune cell refers to the cells produced by humans or animals that have the ability to kill the pathogen and keep the organism healthy.
- Infectivity refers to the ability that one individual has to potentially infect other individuals.
- Individuals who are infected by the infectious disease carry a certain amount of the pathogen. On the contrary, immunity refers to the capability of individuals to resist the pathogen, thereby keeping the organism healthy.
- An infection source is an individual who has infectivity, while susceptible people are the individuals who do not have immunity and have the chance to be infected if in contact with infection sources. Infected individuals are infection sources for cross-infection.
- A superspreader is an individual who carries an unusually large number of pathogens and has sustained infectivity to susceptible individuals within the infection distance. A superspreader is the infection source for direct infection.
- A direct infection (DI) refers to the event where one individual with immune deficiency to a DI contacts the superspreader and becomes infected.
- A cross-infection (CI) refers to the event where one individual with immune deficiency to a CI contacts other infected individuals and becomes infected.
- Individuals have immunity to DI when they contact the superspreader and do not become infected. Individuals have immunity to CI when they contact other infected individuals and do not become infected.

The corresponding DIDM only considers the infection mechanism during epidemic spread. The infection mechanism is mainly formulated based on six infection rules (Rules 1–6). These rules are briefly listed as follows:

Rule 1 (bound of pathogen quantity) The quantity of pathogen carried by each individual is limited. This means the pathogen quantity in each individual has lower and upper limits.
Rule 2 (monotonous characteristic of CI occurrence probability) When an individual has immune deficiency to CI, the more pathogen this individual carries, the worse the health situation of this individual is.
Rule 3 (occurrence probability to direct infection) Regardless of whether an individual with immune deficiency to DI is located within or without the epidemic’s DI zone, there always exists the probability that this individual will become infected by the pathogen or some other cause.
Rule 4 (cross-infection intensity) When an individual carries a greater pathogen amount or there is a shorter distance to their neighbor with immune deficiency to CI, the CI intensity of this individual is larger.
Rule 5 (cross-infection infectivity) An individual must be directly infected before they can infect their neighbors with immune deficiency to CI.
Rule 6 (cross-infection tendency) With CI infectivity increasing or decreasing consistently in some regions, movement of the superspreader away from or closer to these regions will occur gradually. This leads to infectious enhancement or reduction.

Based on the six infection rules, the detailed infection progressing of every individual is represented. However, the DIDM is established mainly based on the infectious mechanism. As for the immune mechanism, it only introduces a prior identical immunity repairing ratio, which hardly represents the natural processes and dynamic behavior in the whole biological immune system. These important, but ignored processes and variation-tendency behavior in the previous work mainly contain immune deficiency, occurrence probabilities, immune cell production, pathogen accumulation, and immune memory mentioned above in the section on the immune mechanism. Therefore, a novel BDIIM is proposed in this work considering both the infectious mechanism and the immune mechanism.

#### 2.2.3. The Proposed Biomimetic Distributed Infection-Immunity Model

Based on the concepts mentioned above in Section 2.2.1, the immune mechanism is given through five rules as follows:

Rule 7 (minimum immune cell amount) Each individual can produce more or less the same number of immune cells; in other words, there exists a normal level of immune cells when the organism is in a healthy state.
Rule 8 (monotonic property of immunity) Each individual agent has the immune ability to produce immune cells. The immune cells are modeled as macrophages, which can devour the pathogens. Therefore, the more immune cells that are produced, the more pathogens that will be killed.
Rule 9 (immune memory) The newly cured individual has an immune memory. Over a period of time, they are unlikely to be infected again. By the end of the memory period, the immune cell number will be decreased to the initial level.
Rule 10 (criterion of immunity deficiency) When determining whether an individual has immune deficiency, the following two conditions must be met: First, the individual is located within the infection distance. Second, the time that the individual is infected is outside of the time of immune memory.
Rule 11 (monotonic property of immune cell rising tendency) The production of immune cells is determined by the amount of newly accumulated pathogen. In other words, the higher the amount of newly accumulated pathogen, the more the organism suffers from the attack of the pathogen. At the same time, more immune cells in the body of the organism will be produced. Furthermore, when the newly accumulated pathogen amount becomes lower, implying that the organism is in a recovery state, more immune cells will be produced, which leads to a rapid decline in the amount of pathogens in the organism.

**Figure 2:**
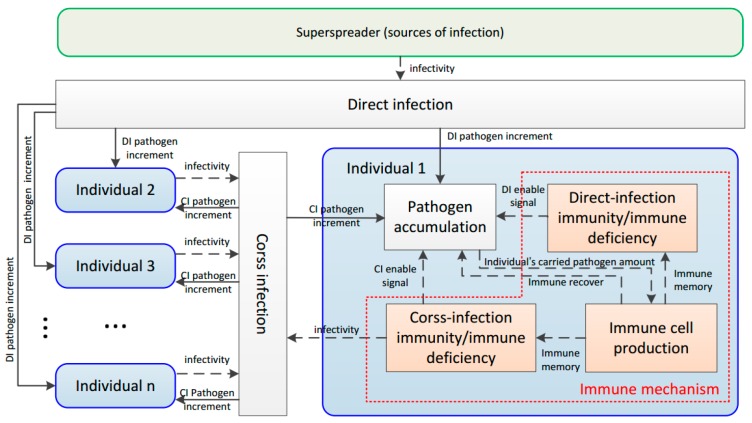
Functional flow process of the proposed biomimetic distributed infection-immunity model (BDIIM) method.

Based on Rules 7–11, the immune response against the invading pathogen is established. The functional flow process of the proposed BDIIM method is given first, as shown in Figure 2. It can be seen that a superspreader, as a source of infection, could infect N individuals simultaneously by direct infection (DI). These individuals experience cross-infection (CI) with each other. For each individual inside this model, there are four functional processes: pathogen accumulation, DI immunity/immune deficiency, immune cell production, and CI immunity/immune deficiency. Based on these concepts and the rules showed above, the proposed algorithm of the BDIIM, composed of six sub-processes, is further established and clarified in the next section.

Some notations for the BDIIM method are also given as follows.

- The individual sets are denoted by *I* = {*I*_1_, ..., *I_k_*, ..., *I_n_*}. The subscript *k* represents the corresponding variable related to the *k*th individual. The distance between individual *I_i_* and *I_j_* is denoted by *D*(*I_i_*, *I_j_*). Denote the distance between the *k*th individual and the superspreader in the *t*th time interval by *d_k_*(*t*). Denote the neighbor collection (including itself) of the *k*th individual by *N_k_* = {*I_j_|D*(*I_j_*, *I_k_*) ≤ *R_c_*, *j ƒ* = *k*}, and *R_c_* is the CI radius.
- A Boolean identification variable *RDp_k_*(*t*) denotes immunity and immune deficiency to DI of the *k*th individual in the *t*th time interval by *RDp_k_*(*t*) = 0 and *RDp_k_*(*t*) = 1, respectively. The immunity and immune deficiency to CI of the *k*th individual in the *t*th time interval is denoted by *RCp_k_*(*t*) = 0 and *RCp_k_*(*t*) = 1, respectively.
- The occurrence probability of DI and CI of the *k*th individual in the *t*th time interval are denoted by *DIp_k_*(*t*) and *CI p_k_*(*t*), respectively. *DIp_k_*(*t*) = 1 happens with probability *P_d_* when the individual is within the epidemic’s DI zone, or with probability *P_f_* when the individual is infected by unknown causes instead of the considered pathogen.
- The event of DI of the *k*th individual in the *t*th time interval is denoted by *φk* (*t*) = 1. The event of CI of the *i*th individual infecting the *j*th individual is denoted by *ϕ_i__→__j_*(*t*) = 1, and the *k*th individual to be infected through CI is denoted by *ϕk*(*t*) = 1. The immune mechanism enable signal of the *k*th individual in the *t*th time interval is denoted by *θk*(*t*) = 1.
- The CI infectivity from the *i*th individual to the *j*th individual at the *t*th time interval is defined by a boolean variable *EC_i→j_*(*t*). *EC_i→j_*(*t*) = 1 and *EC_i→j_*(*t*) = 0 represent “infectivity” and “uninfectivity”, respectively.
- The transmit pathogen amount through DI and CI of the *k*th individual in the *t*th time interval are denoted by *Zd_k_*(*t*) and *Zc_k_*(*t*), respectively. The pathogen amount transmitted from the *i*th individual to the other individuals in the *t*th time interval within the CI zone is denoted by *Z_i_*(*t*).
- The amount of immune cells produced by the *k*th individual in the *t*th time interval is denoted by *M_k_*(*t*). The total amount of immune cells is bound by the maximum and minimum values, *M_max_* and *M_min_*.
- Define the amount of carried pathogen of the individual *I_k_* by *V_k_*(*t*), while *V_max_* and *V_min_* denote the maximum and minimum amounts of carried pathogen, respectively. The newly accumulated pathogen amount is denoted by ∆*V_k_*(*t*).

#### 2.2.4. Six Sub-Processes of the Proposed BDIIM Algorithm

**Figure 3:**
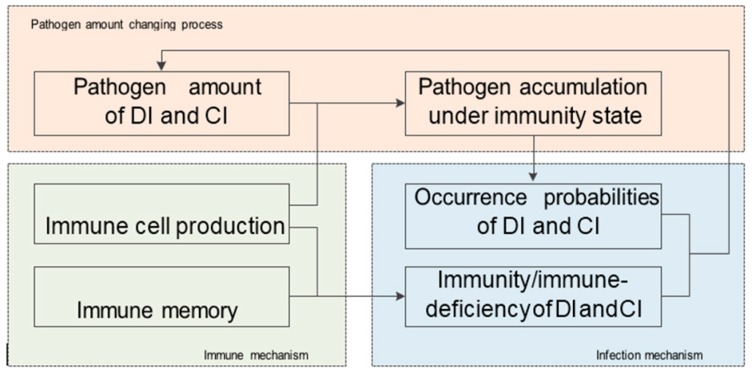
The relationships among the six sub-processes in the BDIIM.

Based on the above concepts and rules, six sub-processes as the core of the proposed algorithm of the BDIIM method are given: the occurrence probabilities of direct-infection and cross-infection, the immunity/immune-deficiency of direct-infection and cross-infection, the pathogen amount of direct-infection and cross-infection, immune memory, immune cell production, and pathogen accumulation under immunity state. The relationships between the six sub-processes of the BDIIM are shown in Figure 3. Further, the sub-processes can be divided into three groups: the infection mechanism, the immune mechanism, and the pathogen amount changing process. The infection mechanism determines whether DI and CI happens or not. The immune mechanism determines the amount of immune cells and whether the individual is immune to the disease or not. The infection mechanism and immune mechanism together control the changing process of the pathogen amounts. The accumulative pathogen amount, as the key parameter in BDIIM, determines the infection probability and the individual’s immunity.

##### Occurrence Probabilities of Direct-Infection and Cross-Infection

When the individual is immune-deficient, they may be infected by the superspreader or other infected individuals. According to Rule 3, each individual infected by DI should be within the DI infectious distance. However, also according to Rule 3, the individuals outside the DI infection zone are also likely to be infected by other unknown factors. Therefore, the occurrence probability of DI (*DIp_k_*) of the *k*th individual is formulated as

(1) $$DIp_{k}(t)=\{\begin{array}{cc} P_{d} & if\begin{array}{c} d_{k}(t)\leq R_{s} \end{array}\\ P_{f} & if\begin{array}{c} \begin{array}{c} d_{k}(t)>R_{s} \end{array} \end{array}\\ 0 & otherwise \end{array}.$$

According to Rule 2, more carried pathogen means a larger infection probability. Therefore, the probability of CI (*CIp_k_*) of the *k*th individual is formulated as

(2) $$CIp_{k}(t)=f_{c}(\begin{array}{cccc} V_{k}(t), & \Delta V_{k}(t-1)|V_{\min}, & V_{\max}, & \mu \end{array})$$

where *f_c_*(·) denotes the general probability function. The probability function monotonously increases with the newly accumulated pathogen amount ∆*V_k_*(*t*) and the carried pathogen amount *V_k_*(*t*). The initial value is set as ∆*V_k_*(0) = 0. The function parameters *V_min_* and *V_max_* denote the upper and lower bounds, respectively. The medical isolation parameter *µ* ∈ [0, 1] denotes the CI propagation degree. *µ* = 0 means that susceptible individuals are absolutely isolated from the infected individual, and *µ* = 1 represents that no isolation countermeasures are taken for susceptible individuals.

**Remark** **1.** *The occurrence of probability is described as the immune deficiency probability in our previous work. Nevertheless, this description is not sufficient. It is common sense, and is also shown in Rule 10, that the susceptible individual should satisfy two conditions: the individual is immune-deficient, and this individual is within the infectious distance. However, as a matter of fact, not all susceptible individuals are infected after contacting infection sources. Therefore, the immune deficiency probability is confused with the infection probability. In this work, both are redefined: the probability that one individual is susceptible is formulated as the probability of immune deficiency, while the probability that one individual is infected after contacting the infection source is formulated as the infection occurrence probability.*

**Remark** **2.** *The probabilities described in Equation* (*2*) *are not unique; they only represent a general occurrence probability model about infectious disease. Users can design different occurrence probability functions for different kinds of disease via Equation* (*2*).

**Remark** **3.** *The probability DIp_k_(t) depends on the superspreader’s carried pathogen amount and the distance between the kth individual and the superspreader. In this work, both are considered to be unknown. Therefore, a priori knowledge P_d_ and P_f_ are used to formulate DIp_k_(t).*

##### Immunity/Immune-Deficiency of Direct-Infection and Cross-Infection

According to Rule 2, a greater amount of carried pathogen indicates a worse health situation, so the probability of immune-deficiency of DI of *I_k_* at the *t*th time interval is formulated as follows:

(3) $$RDp_{k}(t)=f_{d}(\begin{array}{ccc} V_{k}(t), & \Delta V_{k}(t-1)|V_{\min}, & V_{\max} \end{array}).$$

*I_k_* will be infected through CI by *I_i_* if three conditions are satisfied. First, *I_i_* has infectivity. In other words, according to Rule 5, *I_i_* is infected through DI. Second, according to Rule 10, the distance between *I_i_* and *I_k_* is within the infectious distance of *I_i_*. Third, according to Rule 10, *I_k_* is out of immune memory. Taking all three conditions into consideration, the probability of immune-deficiency of CI from *I_i_* to *I_k_* in the *t*th time interval is formulated as follows:

(4) $${\mathrm{RC}}_{i\to k}(t)=\{\begin{array}{cc} 1 & if\begin{array}{c} \left(EC_{i\to k}(t)=1)\cap (D(I_{i},I_{k})\leq R_{c})\cap (IMMT_{k}(t)=0\right) \end{array}\\ 0 & otherwise \end{array}$$

where *IMMT_k_*(*t*) denotes the immune memory of the *k*th individual at the *t*th time interval. Its definition will be detailed later.

According to Rule 5, the cross-infectivity is defined as

(5) $$EC_{i\to k}(t)=\{\begin{array}{cc} 1 & \phi_{i}(t)=1\\ 0 & otherwise \end{array}$$

**Remark** **4.** *It is worth mentioning that RC p_i→k_(t) represents the immunity of an individual to CI. One important condition of CI occurring is immune deficiency; however, this was ignored in our previous work, which leads to the event that some individuals residing outside of the infectious distance should be considered as immune-deficient.*

##### Pathogen Amount of Direct-Infection and Cross-Infection

Taking Equations (1) and (3) into consideration, the probability of *DI* occurrence is formulated as

(6) $$P\left\{\phi_{k}(t)=1\right\}=DIp_{k}(t)\cdot RDpk(t)$$

The transmit pathogen amount of *DI* is defined as a constant *κ_d_*, then in the *t*th time interval, the pathogen amount obtained through *DI* is defined as

(7) $$Zd_{k}=\phi_{k}(t)\cdot \kappa_{d}$$

Taking Equations (2) and (4) into consideration, the *CI* occurrence probability from the *i*th individual to the *k*th individual is formulated as

(8) $$P\left\{\varphi_{i\to k}(t)=1\right\}=CIp_{k}(t)\cdot RCp_{i\to k}(t)\cdot EC_{i\to k}(t)$$

Before defining the transmit pathogen amount of *CI*, according to Rule 4, we first construct the cross-infection infectivity level (CIIL) as follows:

(9) $$CIIL_{k}(t)=\sum_{j\in N_{k},EC_{j\to k}=1}\frac{V_{j}(t)/V_{\max}}{D(I_{j},I_{k})/R_{c}}$$

where *D*(*I_i_*, *I_k_*)/*R_c_* and *V_j_*(*t*)/*V_max_* are the normalized distance and normalized pathogen amount, respectively. According to Rule 6, the cross-infection intensity factor (CIIF) is constructed as follows:

(10) $$CIIF_{k}(t)=\{\begin{array}{cc} c_{1} & CIIL_{k}(t-2)\leq CIIL_{k}(t-1)\leq CIIL_{k}(t)\\ c_{3} & CIIL_{k}(t-2)>CIIL_{k}(t-1)>CIIL_{k}(t)\\ c_{2} & else \end{array}.$$

According to Rules 5 and 10, each individual with infectivity may infect their neighbors with immune deficiency, and the pathogen amount corresponding to the individual’s neighbors increases. The pathogen increment increases monotonically with CIIF and the infective individual’s carried pathogen amount. The pathogen increment is described as follows:

(11) $$Z_{k}(t)=\frac{V_{k}(t)-V_{\min}}{V_{\max}-V_{\min}}CIIF_{k}(t).$$

Taking Equation (11), the released pathogen amount *Z_k_*(*t*) is proportionate to the carried pathogen amount. If *V_k_*(*t*) = *V_max_*, *Z_k_*(*t*) will be largest; if *V_k_*(*t*) = *V_min_*, *Z_k_*(*t*) will be zero, so CI will not occur. In the *t*th time interval, all the pathogen amounts obtained through CI are defined as

(12) $$Zc_{k}(t)=\sum_{i\in N_{k}}\varphi_{i\to k}(t)\cdot Z_{i}(t).$$

The event that the *k*th individual is infected by CI is defined as

(13) $$\varphi_{k}(t)=\{\begin{array}{ccc} 1 & \begin{array}{cc} if & \end{array} & Zc_{k}(t)>V_{\max}\\ 0 & otherwise & \end{array}.$$

##### Immune Cell Production

To formulate the immune cell production tendency, the immunity intensity factor (IMMF) is first defined according to Rule 11:

(14) $$IMMF_{k}(t)=\{\begin{array}{cc} IMMF_{k}(t)+m_{1}\Delta V_{k}(t) & if\begin{array}{c} \left(\phi_{k}(t)=1)\cap (\Delta V_{k}(t)\geq 0\right) \end{array}\\ m_{2}IMMF_{k}(t) & if\begin{array}{c} \left(\phi_{k}(t)=1)\cap (\Delta V_{k}(t)<0\right) \end{array}\\ m_{3}IMMF_{k}(t) & if\begin{array}{c} \left(\phi_{k}(t)=0)\cap ((RDp_{k}(t)\cup RCp_{i\to k}(t))=1\right) \end{array}\\ m_{4} & otherwise \end{array}$$

where *I* ∈ *N_k_* and *m*_1_–*m*_4_ are immune coefficients, with *m*_1_ < *m*_2_ < *m*_3_. According to Rule 7, the immune cell amount is given as follows:

(15) $$M_{k}(t)=\{\begin{array}{cc} M_{\min} & if\begin{array}{c} M_{k}(t)\leq M_{\min} \end{array}\\ IMMF_{k}(t)\cdot M_{k}(t) & if\begin{array}{c} M_{\min}<M_{k}(t)<min(M_{\max},V_{k}(t)) \end{array}\\ M_{\max} & if\begin{array}{c} M_{k}(t)\geq min(M_{\max},V_{k}(t)) \end{array} \end{array}.$$

Whether an immune cell is produced or not is highly related to the individual’s newly accumulated pathogen amount. When the individual agents are in the early phase of the mode of *ϕk*(*t*) = 1, the number of immune cells is small and the pathogen amount increases rapidly. In the last phase of *ϕk*(*t*) = 1, if the immune cell number is larger than the pathogen amount, then the corresponding individual will be recovered from infectious disease.

##### Immune Memory

According to Rule 9, immune memory can be formulated as

(16) $$IMMT_{k}(t)=\{\begin{array}{cc} m_{5} & if\begin{array}{c} \left(\phi_{k}(t-1)\cap (\phi_{k}(t)=0\right) \end{array}\\ IMMT_{k}(t-1)-1 & if\begin{array}{c} IMMT_{k}(t-1)\neq 0 \end{array}\\ 0 & otherwise \end{array}$$

where *m*_5_ ≥ 0 denotes the time length of the immune memory.

##### Pathogen Accumulation under Immunity State

The pathogen amount changes due to three sources: the newly accumulated pathogen amount due to DI and CI, ∆*V_k_*(*t*), the residue of the previously accumulated pathogen amount, *V_k_*(*t* − 1), and the decrease in the pathogen amount due to immunity repair, *M_k_*(*t*). The immune enable signal is given in Equation (17), which refers to the immune mechanism occurring in the *k*th individual if they are infected through CI or DI in the *t*th time interval:

(17) $$\theta_{k}(t)=\{\begin{array}{cc} 1 & if\begin{array}{c} \left(\phi_{k}(t)=1\cap (\underset{i\in N_{k}}{\cup }\varphi_{i\to k}(t)=1\right) \end{array}\\ 0 & otherwise \end{array}.$$

Hence, the accumulated pathogen amount based on Rule 1 is

(18) $$V_{k}(t)=\min\left\{V_{\max},\max\left\{V_{k}(t-1)+\Delta V_{k}(t)-\theta_{k}(t)\cdot M_{k}(t)，V_{\min}\right\}\right\}.$$

With the Boolean variable *RC_i_*_→*k*_ (*t*), the newly accumulated pathogen amount is

(19) $$\Delta V_{k}(t)=Zd_{k}(t)+Zc_{k}(t).$$

Then, the newly accumulated pathogen amount ∆*V_k_*(*t*) and the accumulated pathogen amount *V_k_*(*t*) measure the probability of superspreader existence and, as shown in Equations (2) and (3), determine the occurrence probability of CI and the probability of immune-deficiency to DI in the next time interval.

## 3. The Corresponding Relationship between the Proposed BDIIM and Node Wake-Up Process

In this section, similarities between sensing nodes in sensor networks and individuals in epidemic areas are established.

- Collaboration between the self-organized sensor nodes is similar to the infectious interactions between these individuals.
- A sensor node obtains the target information using the SM or CM while an individual is infected through DI or CI.
- For the sensor nodes in WSNs, when a sensor node finds a target using its SM, this node then communicates with its neighbors via a CM. For the individuals in an epidemic area, when an individual is infected by DI, this individual then propagates the pathogen to their neighbors by CI.
- Using a sensor node SM in a “wake” state, it is possible to find the target, while DI of an individual with immune deficiency may occur with the presence of the superspreader.
- A sensor node CM in the sleeping state cannot obtain target information, while CI of an individual with immunity does not occur with their neighbors.

Based on these similarities between the BDIIM and the sensor node wake-up process, parameter correspondence between BDIIM and sensor nodes are established, as listed in Table 1. Further, for the *k*th individual and corresponding *k*th sensor node, the corresponding activity relationships between them are listed in Table 2.

**Figure 4:**
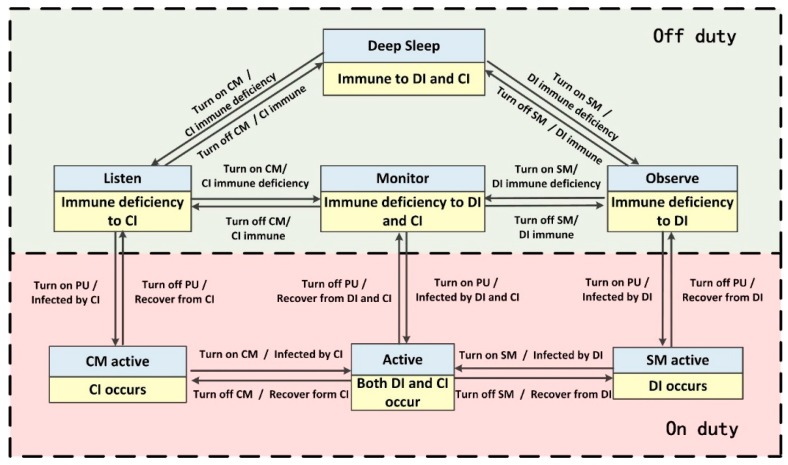
The corresponding relationships between the BDIIM and sensor wake-up control (WC) states.

Corresponding relationships composed of seven pairs of states between BDIIM and the sensor’s WC state are shown in Figure 4. Each pair of states contains a state of the BDIIM and a WC state marked in a yellow and gray box, respectively. Each pair of states denotes the corresponding relationships between the BDIIM and the sensor’s WC states. According to the above proposed algorithm in Section 2 and the settings in this section, when a target appears, all of these seven groups of states will transform continuously to achieve wake-up control of each sensor node. There are two modes for the sensors: on duty and off duty. When the sensor is in “on duty” mode, the processing unit is powered on, at least one of the SM and/or CM is powered on, and the sensor is capable of processing the sensory messages. On the contrary, when the sensor is in “off duty” mode, the processing unit (PU) is powered off, and the SM and CM are either turned off or turned on to wait for wakeup signals. The corresponding relationships between the WC states and sensors states are listed in Table 3.

## 4. Numerical Example

The simulation was formulated in Matlab R2012a and the initial conditions were set as follows: Five hundred micro-sensors are assumed to be randomly planted on the ground in the square region of 40,000 square meters. These sensors integrate CMs receiving and sending modules, and the SMs can detect the target and transfer the target information via the CMs when the target passes by. The time interval was 1 s, and the detailed variables are listed in Table 4.

The initial values are ∆*V_k_*(0) = 0 and *V_k_*(0) = *V*_min_. *CIp_k_*(*t*) and *RDp_k_*(*t*) in Equations (2) and (3) are considered as

(20) $$CIp_{k}(t)=RDp_{k}(t)=\{\begin{array}{cc} V_{k}(t)/V_{\max} & if\begin{array}{c} \Delta V_{k}(t-1)\neq 0 \end{array}\\ V_{\min}/V_{\max} & if\begin{array}{c} \Delta V_{k}(t-1)=0 \end{array} \end{array}.$$

**Figure 5:**
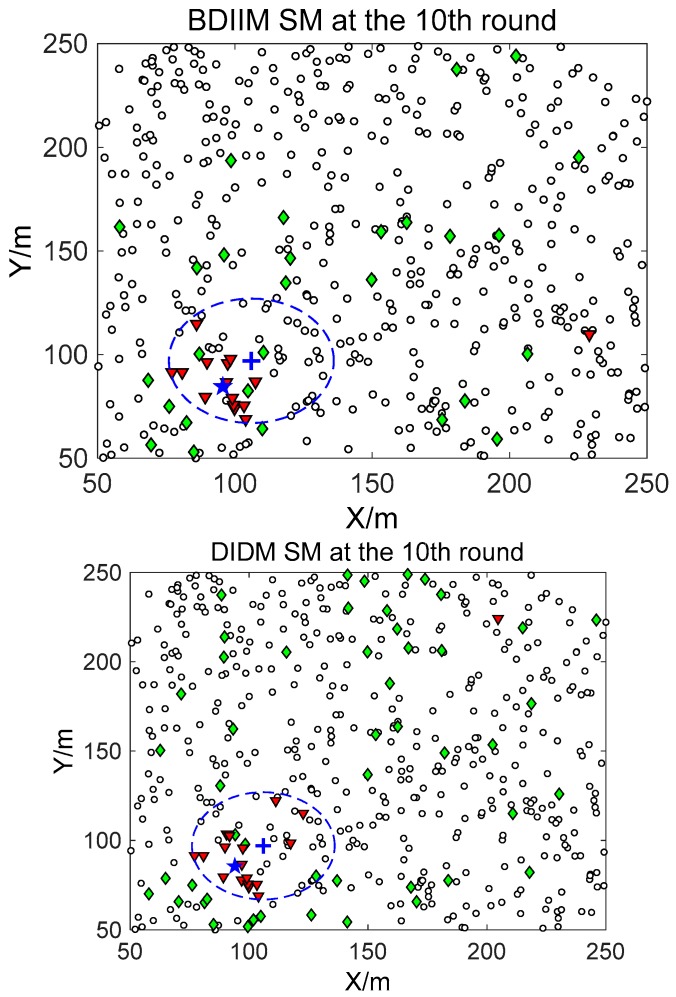
The state of the sensing module (SM) at the 10th time interval.

**Figure 6:**
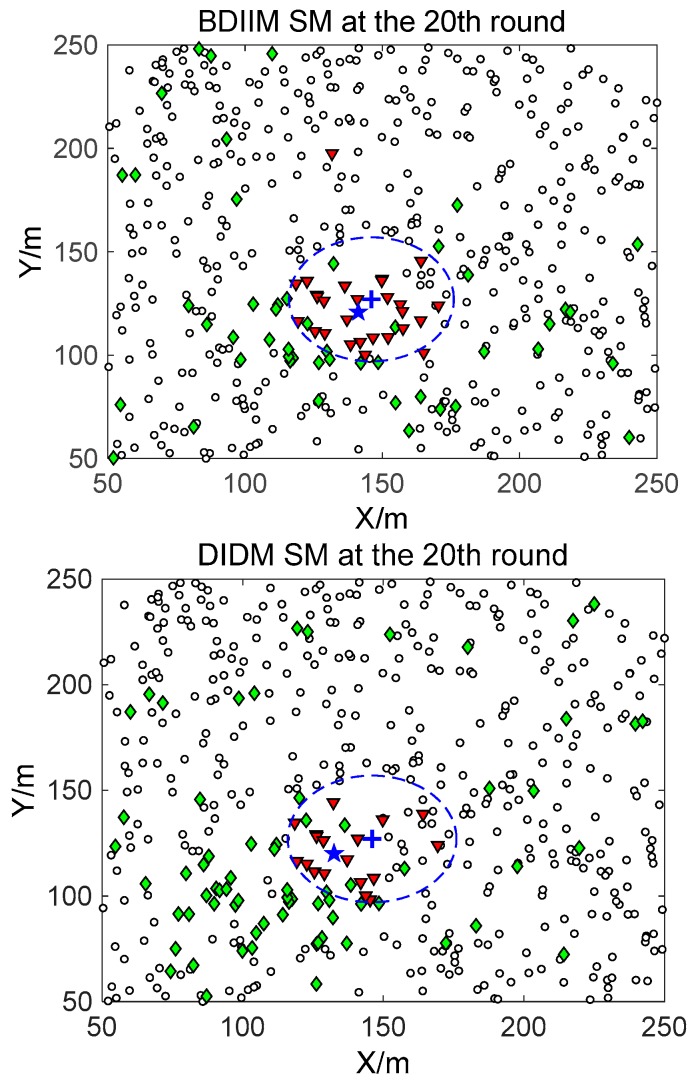
The state of the SM at the 20th time interval.

**Figure 7:**
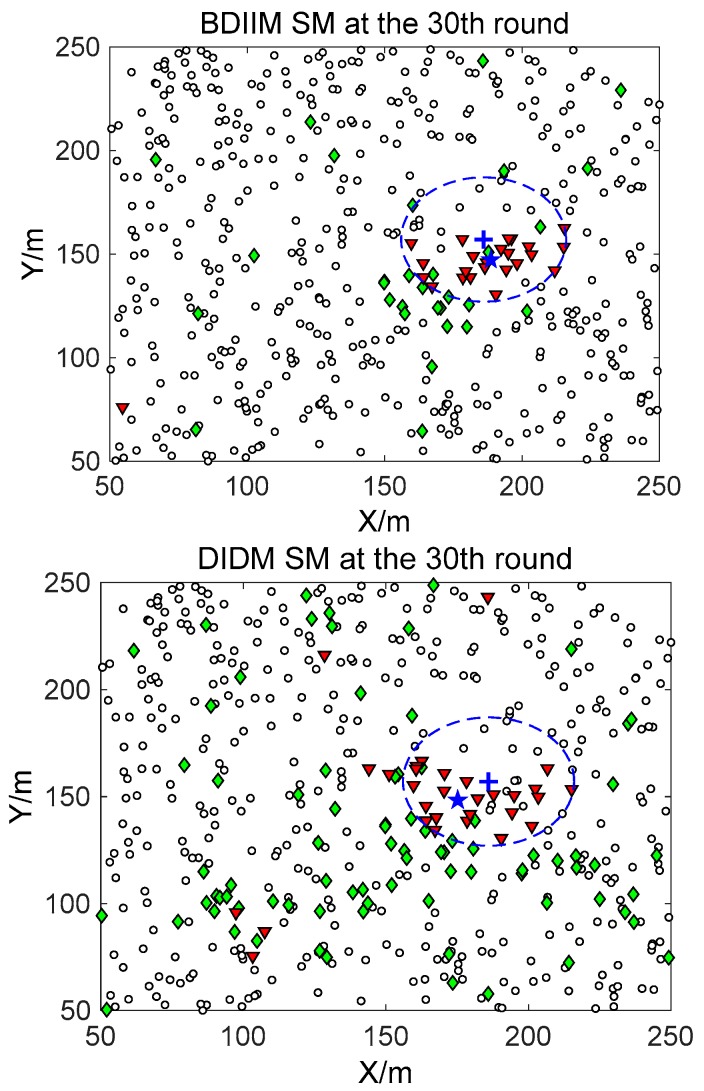
The state of the SM at the 30th time interval.

The target moves in constant-velocity (CV) driven by a zero-mean Gaussian noise process with a variance of 400 m^2^ in 10 consecutive rounds. Figure 5, Figure 6 and Figure 7 show in detail the state of the SMs at different times during the target movement via the BDIIM and DIDM methods. The target is plotted by a “**+**” (blue), which is supposed to have the same track in both methods and move from the left-lower location of the figure to an upper-right location. “★” (blue) represents the estimated target position. The dashed circle (blue) denotes the sensing and communicating area with the radii *R_s_* and *R_s_* + *R_c_*, respectively. “**□**” (black) in the smallest size represents the “sleep” SMs. “▼” (red) represents the valid wake-up SMs, which are set to “wake” and successfully find the true target. “◆” (green) represents the invalid wake-up SMs, which are set to “wake” but fail to find the target. The SM states in the 10th, 20th, and 30th time intervals are compared. The results in the other time intervals are similar and are therefore saved. In counting the total number of green “◆” outside the circle at these three time intervals, we find that the number of green “◆” via the proposed BDIIM method is slightly less than the number obtained via the previous DIDM method. Meanwhile, in the area outside the circles where the target passes by, the number of green “◆” via BDIIM is much less than that achieved via DIDM.

**Figure 8:**
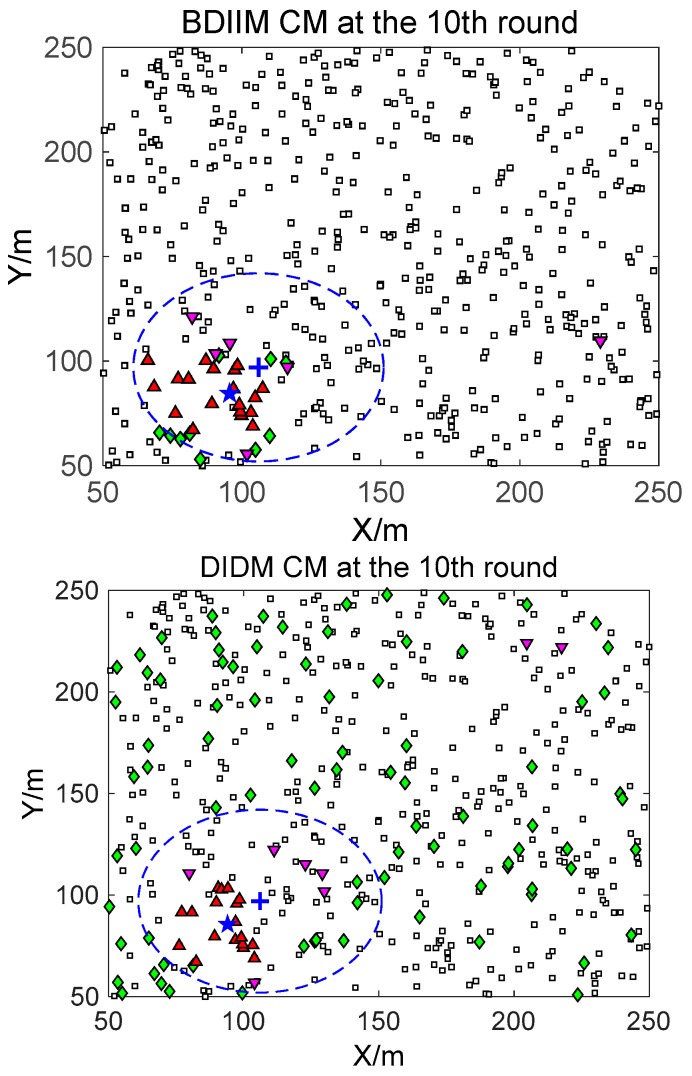
The state of the communication module (CM) at the 10th time interval.

**Figure 9:**
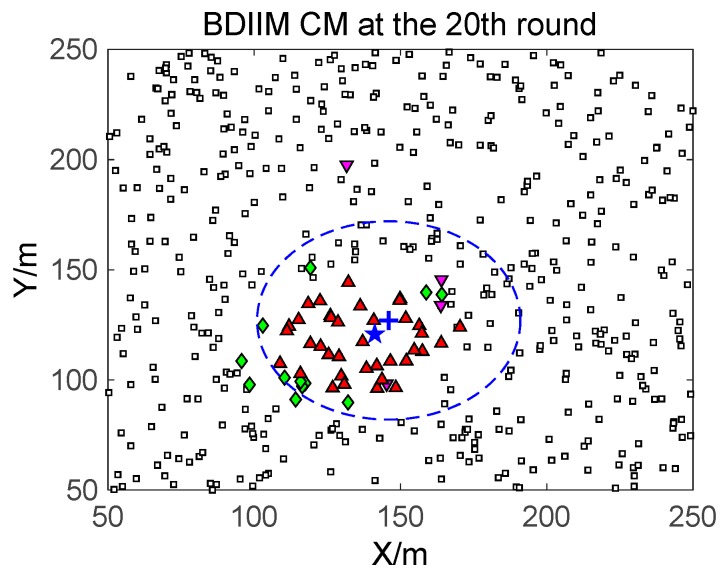

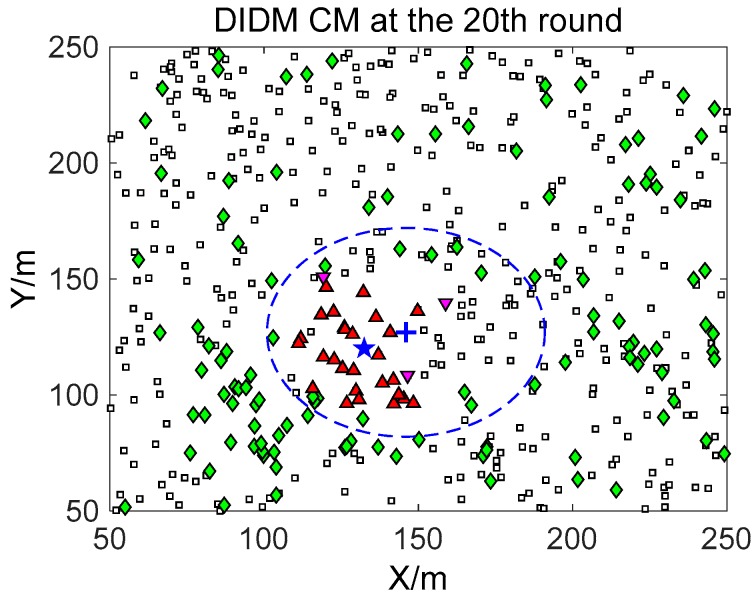
The state of the CM at the 20th time interval.

**Figure 10:**
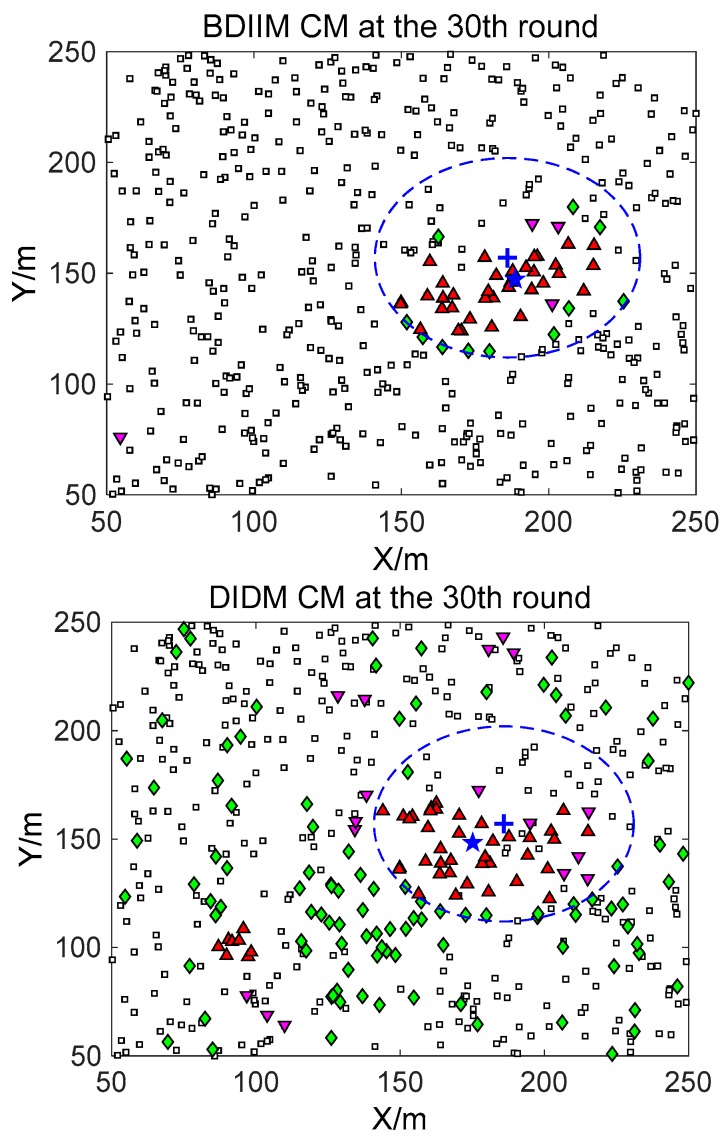
The state of the CM at the 30th time interval.

For the state of the CMs, as shown in Figure 8, Figure 9 and Figure 10, the target is plotted by a “+” (blue). “▲” (red) represents the valid wake-up CMs. “▼” (pink) represents the invalid wake-up CMs in which “◆” (green) represents the invalid wake-up CMs in the “send/receive” state. These figures show the detailed states of the CMs at the 10th, 20th, and 30th time intervals during target movement, via the BDIIM and DIDM methods. Especially in the area outside the circles, where the target passes by, the number of green “◆” via the BDIIM is clearly much less than that obtained via the DIDM at each time interval. In other words, the proposed BDIIM method has a higher wake-up efficiency than the DIDM method, and the energy cost of the system is greatly reduced, while the estimation error of the target position also decreases.

**Figure 11:**
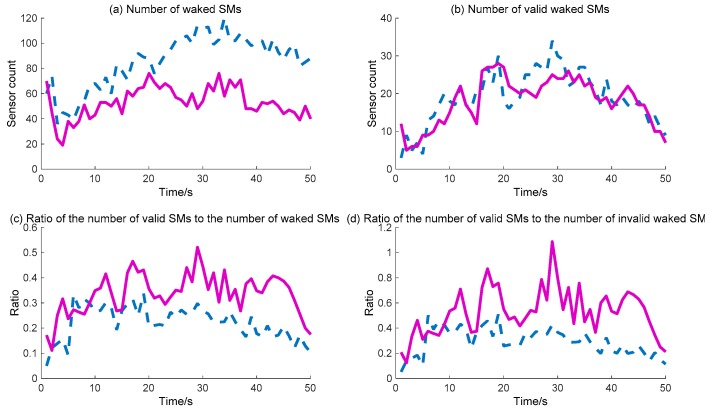
The WC performance of SMs: (**a**) Number of waked SMs. (**b**) Number of valid waked SMs. (**c**) Ratio of the number of valid SMs to the number of waked SMs. (**d**) Ratio of the number of valid SMs to the number of invalid waked SMs. (**—**: BDIIM; --: distributed infectious disease model (DIDM)).

**Figure 12:**
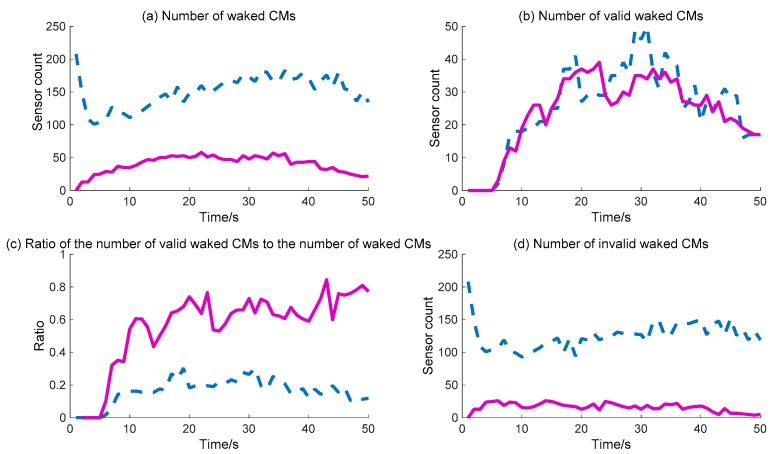
The WC performance of CMs: (**a**) Number of waked CMs. (**b**) Number of valid waked SMs. (**c**) Ratio of the number of valid waked CMs to the number of waked CMs. (**d**) Number of invalid waked CMs. (**—**: BDIIM; --: DIDM).

Further, the detailed data regarding the DIDM method and the proposed BDIIM method are curved, as shown in Figure 11 and Figure 12. The dashed (blue) and solid (pink) lines represent the DIDM method and the proposed BDIIM method, respectively. The WC performance of the SMs is shown in Figure 11. The detailed calculation data are listed in Table 5. It can first be seen from Figure 11b that, throughout the whole process, there is little difference in the number of valid waked SMs between the two methods. However, it can be clearly seen from Figure 11a that the number of waked SMs via the BDIIM method is fewer than that achieved via the DIDM method throughout the whole process, especially after 20 s. The ratio of the number of valid waked SMs to the total number of waked SMs is given in Figure 11c. The average ratio via the proposed method in this work approaches 0.38, while the average ratio via the previous method is about 0.25 during this period. Compared with the previous method, the ratio is improved by approximately 52% by using the proposed method. Furthermore, the ratio of the number of valid waked SMs to the idle number of waked SMs, shown in Figure 11d, demonstrates that the wake efficiency of SMs via the BDIIM method is higher than that achieved via the DIDM method.

The WC performance of the CMs is shown in Figure 12. The detailed calculation data are listed in Table 6. Figure 12b shows that the number of valid waked CMs is no different across the whole process for the two methods, while Figure 12a shows that the number of waked CMs via the BDIIM is clearly less than that achieved via the DIDM method. During the whole process, the maximum number of waked CMs via the BDIIM method is approximately 50 after 10 s, while the maximum number via the DIDM method reaches more than 150 much more rapidly. Naturally, the ratio of the number of valid waked CMs to the total number of waked CMs demonstrated by the BDIIM method is much higher than that proposed by the DIDM method, as shown in Figure 12c, which means that the efficiency of the waked CMs is improved greatly using the BDIIM method. The average efficiency after 10 s using the BDIIM method is estimated to be approximately 65%, compared with an average efficiency of just 21% achieved by the DIDM method. In addition, the number of idle sensor nodes is also given in Figure 12d, and it can be seen that the average number of idle nodes is 15 in the BDIIM method, which is much less than the 127 that would occur using the DIDM method. The percentage of the number of invalid waked CMs for the BDIIM method compared with the DIDM method is just 12.4%.

**Figure 13:**
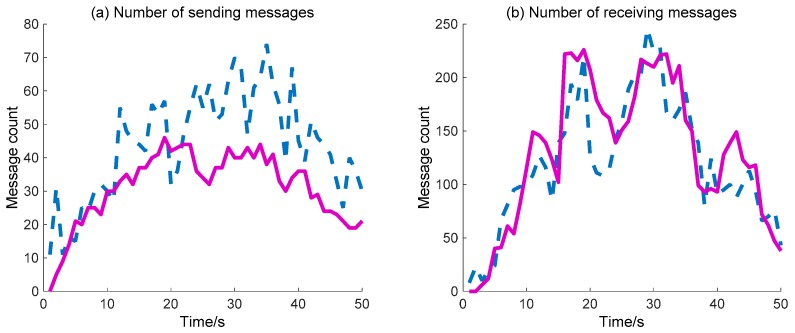
The WC performance of the sending and receiving messages numbers: (**a**) Number of sending messages. (**b**) Number of receiving messages. (**—**: BDIIM; --: DIDM).

The sending/receiving message number is also considered to compare the WC performance of the two methods (DIDM and BDIIM), as shown in Figure 13. The detailed calculation data are listed in Table 7. The sending messages number achieved via the proposed BDIIM method is lower than that achieved via the previous DIDM method. According to the given ratio of receiving messages to sending messages, representing the efficiency of the WC, the improvement in time of the BDIIM method is 1.59 s when compared with the DIDM method.

**Figure 14:**
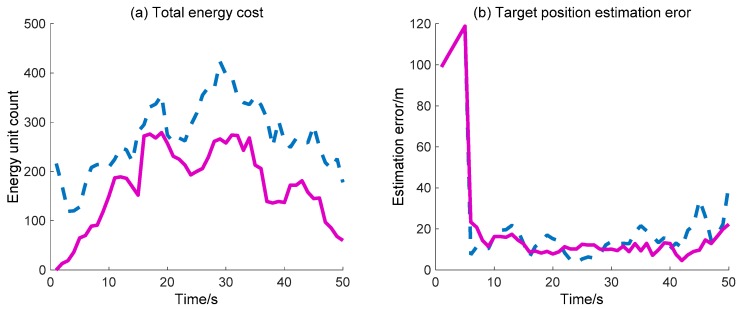
Energy cost and target position estimation in WC performance: (**a**) Total energy cost. (**b**) Target position estimation error. (**—**: BDIIM; --: DIDM).

The target position estimation error and total energy cost of WSN are also simulated and compared with the previously mentioned DIDM method. Here, the target position estimation error denotes the distance between the actual target position and the estimate position (the centroid of the valid waked sensor nodes). As for the total energy cost, we consider that the energy consumed by the “idle” sensor node in each time interval is one unit, and one sensor node sending/receiving a message is also considered one unit. Due to the fact that the calculation operation costs much less energy compared with the operation of sending/receiving messages, the calculation cost is not considered. In Figure 14, the total energy cost across the whole process via the proposed BDIIM method is always less than that obtained via the DIDM method. Finally, the target position estimation error is basically consistent during the first 30 s, while the error via the proposed BDIIM method is slightly less than that obtained via the DIDM method after 30 s. Finally, the total energy cost and position estimation error of the BDIIM method, as listed in Table 8, are reduced to 50.8% and 78.9% compared with the DIDM method, respectively.

## 5. Conclusions

Inspired by the immune mechanisms in multicellular organisms, a biomimetic distributed infection-immune model (BDIIM) used for the purpose of wake-up control in multi-sensor fusion has been proposed. The proposed BDIIM is largely composed of six sub-processes: the occurrence probabilities of DI and CI, the immunity/immune-deficiency of DI and CI, the pathogen amount of DI and CI, immune cell production, immune memory, and pathogen accumulation under immunity state. Furthermore, by establishing the corresponding relationships between sensor wake-up and infectious disease propagation, the proposed BDIIM is utilized to derive a collaborative sensor wake-up strategy. The simulation we ran showed that BDIIM can reach a better cost-effective tradeoff between accurate target location and low energy consumption. The energy consumption of both SMs and CMs significantly decreases. Finally, the total energy cost and position estimation error of the proposed BDIIM method are reduced to 50.8% and 78.9%, respectively.

## Figures and Tables

**Table 1:** Parameter correspondence between the BDIIM and the sensor nodes.

Parameter of WC	Parameter of BDIIM Model	Symbol
Target	Epidemic superspreader	
*k*th sensor node	*k*th individual	*I_k_*
Sensing range	DI radius	*R_s_*
Communication range	CI radius	*R_c_*
Probability that a waked node detects the target within sensing range	Probability that an individual with immune deficiency to DI within the epidemic’s DI zone is infected by the superspreader	*P_d_*
Probability that a node falsely confirms the existence of the target	Probability that an individual is infected by an unknown cause instead of the superspreader	*P_f_*

**Table 2:** Corresponding activity relationships between the *k*th node and the individual.

Activity in WC	Activity in BDIIM Model	Denotation
*k*th sensor node finds the target based on SM	DI occurs	*ϕ_k_*(*t*) = 1
*i*th sensor node communicates the target information to the *j*th node	*i*th individual infects *j*th individual	*EC*_i→j_(*t*) = 1
*i*th node confirms the existence of the target based on information from *j*th node	CI from *i*th individual to *j*th individual occurs	*φ*_i→j_(*t*) = 1
*k*th node finds the target from both SM and CM	Both CI and DI occur	*ϕ_k_*(*t*) = 1 and *φ_k_*(*t*) = 1
*k*th node’s SM is in sleep mode	*k*th individual is immune to DI	*RDp_k_*(*t*) = 1
*k*th node’s SM is in idle mode	*k*th individual has immune deficiency to DI	*RDp_k_*(*t*) = 0
*k*th node’s SM is in wake mode	*k*th individual is infected by DI	*ϕ*_k_(*t*) = 1
*k*th node’s CM is in sleep mode	*k*th individual is immune to CI	*RCp_i_*_→k_(*t*) = 1 and *i*∈*N_k_*
*k*th node’s CM is in idle mode	*k*th individual has immune deficiency to CI	*RCp_i_*_→k_(*t*) = 0 and *i*∈*N_k_*
*k*th node’s CM is in receive/send mode	*k*th individual is infected by their neighbors through CI	*φ_k_*(*t*) = 1

**Table 3:** The corresponding relationships between the WC states and sensor states.

Mode	WC State	Sensor State		
		SM	CM	PU
Off duty	deep sleep	Sleep	Sleep	Sleep
	observe	Idle	Sleep	Sleep
	listen	Sleep	Idle	Sleep
	monitor	Idle	Idle	Sleep
On duty	SM active	Wake	Sleep	Wake
	CM active	Sleep	Send/receive	Wake
	active	Wake	Send/receive	Wake

**Table 4:** Variable settings.

Var	Value	Var	Value	Var	Value	Var	Value	Var	Value	Var	Value	Var	Value
*R_S_*	30*m*	*P_d_*	0.9	*V*_max_	20	*c*_1_	8	*c*_3_	0	*m*_2_	1.2	*m*_4_	1
*R_c_*	15*m*	*P_f_*	0.05	*V*_min_	1	*c*_2_	4	*m*_1_	0.25	*m*_3_	2	*m*_5_	3

**Table 5:** Data of the SM performance of nodes in WC.

	Number of Waked SMs	Number of Valid Waked SMs	Ratio of Number of Valid SMs to the Number of Waked SMs
DIDM method	81	22	25%
BDIIM method	49	19	38%
Improvement time	/	/	1.52

**Table 6:** Data of the CMs performance of nodes in WC.

	Number of Waked CMs	Number of Valid Waked CMs (after 10 s)	Ratio of Number of Valid CMs to the Number of Waked CMs (after 10 s)	Number of Invalid Waked CMs
DIDM method	153	32	21%	121
BDIIM method	42	27	65%	15
Improvement time	/	/	3.1	12.4%

**Table 7:** The numbers of sending and receiving messages nodes in the WC.

	Average Number of Sending Messages	Average Number of Receiving Messages	Ratio of the Receiving to the Sending
DIDM method	52	145	2.79
BDIIM method	33	147	4.45
Improvement time	/	/	1.59

**Table 8:** Energy cost and position estimation error of the nodes in WC.

	Total Energy Cost	Target Position Estimation Error (after 10 s)
DIDM method	297	19.5
BDIIM method	151	15.4
Reduction percentage	50.8%	78.9%

## Abbreviations

The following abbreviations are used in this manuscript:

AAC	artificial ant colony
CM	communication module
CI	cross-infection
DIDM	distributed infectious disease model
BDIIM	biomimetic distributed infection-immunity model
DI	direct infection
PU	processing unit
SM	sensing module
WSN	wireless sensor network
WC	wake-up control
